# Construction and analysis of the protein-protein interaction network related to essential hypertension

**DOI:** 10.1186/1752-0509-7-32

**Published:** 2013-04-12

**Authors:** Jihua Ran, Hui Li, Jianfeng Fu, Ling Liu, Yanchao Xing, Xiumei Li, Hongming Shen, Yan Chen, Xiaofang Jiang, Yan Li, Huiwu Li

**Affiliations:** 1 Basic Medicine School, Xinjiang Medical University, Urumqi, Xinjiang, China; 2 Clinical Laboratory Diagnosis Center of PLA, Urumqi General Hospital of Lanzhou Command, Urumqi, China; 3 393 Xinyi Rd, Urumqi, Xinjiang, 830011, P.R. China

**Keywords:** Protein-protein interaction (PPI), Network, Essential hypertension

## Abstract

**Background:**

Essential hypertension (EH) is a complex disease as a consequence of interaction between environmental factors and genetic background, but the pathogenesis of EH remains elusive. The emerging tools of network medicine offer a platform to explore a complex disease at system level. In this study, we aimed to identify the key proteins and the biological regulatory pathways involving in EH and further to explore the molecular connectivities between these pathways by the topological analysis of the Protein-protein interaction (PPI) network.

**Result:**

The extended network including one giant network consisted of 535 nodes connected via 2572 edges and two separated small networks. 27 proteins with high BC and 28 proteins with large degree have been identified. NOS3 with highest BC and Closeness centrality located in the centre of the network. The backbone network derived from high BC proteins presents a clear and visual overview which shows all important regulatory pathways for blood pressure (BP) and the crosstalk between them. Finally, the robustness of NOS3 as central protein and accuracy of backbone were validated by 287 test networks.

**Conclusion:**

Our finding suggests that blood pressure variation is orchestrated by an integrated PPI network centered on NOS3.

## Background

Hypertension is a main risk factor of stroke, heat failure and ischemic heart disease. In spite of the huge amount of researches recently performed in this area, the pathogenesis of human hypertension remains elusive. Thus, hypertension has to be defined as “essential” for 95 to 99% of cases [1]. Essential hypertension (EH) is viewed as a consequence of interaction between environmental factors and genetic background. Data from animal models, human twin and family studies have indicated that approximately 30%-60% of BP variation is caused by genetic factors [2,3]. Furthermore, association study and linkage analysis have determined many casual or susceptible genes related to EH. BP must be a highly regulated quantity, affected by a multitude of physiological systems that finally integrate and maintain BP levels to secure an adequate blood perfusion of all tissues [4]. BP variation is a consequence of altered activity in signal transduction pathways and interactions of complex intra- and intercellular processes. As all biochemical processes are governed by the proteins, we propose that protein–protein interactions (PPIs) especially the proteins encoded by these casual or susceptible genes are extremely important in orchestrating the BP variation.

In the recent years, the topological analyses have been applied to molecular networks including protein interaction networks, whose nodes are proteins linked to each other via physical interactions [5]. In this study, we aimed to identify the important proteins and the biological regulatory pathways involving in EH and further explore the molecular connectivity between these pathways by the topological analysis of the PPIs network derived from the proteins encoded by casual or susceptible genes for EH. The parameters of degree and betweenness are two fundamental measures in network theory. Degree measures how many neighbors a node direct connect to while betweenness measures how often nodes occur on the shortest paths between other nodes [6]. In the PPIs network the nodes with high degree defined as hub protein and the nodes with high betweenness defined as bottleneck protein, both are key or important protein [6]. Yu H and colleagues think of protein networks in analogy to a transportation network, so proteins with high betweenness are similar to heavily used intersections, such as those leading to major highways or bridges [7]. In this study, we employed the proteins with high BC to identify the important genes and their related signal pathways in regulating BP.

## Methods

The research method used in this study mainly consisted of seven steps. Step one: extraction of the candidate genes associated with EH from the literature using PolySearch text mining system. Step two: Scanning protein interactions from the database STRING. Step three: Construction of PPIs network and extraction the giant component from the extended network. Step four: Topological Analysis of PPI network. Step five: extraction the large BC nodes from the giant network to create a backbone network. Step six: Construction a subnetwork consisting of all shortest paths between the candidate genes from the giant network. Step seven: Validation of the backbone network and the NOS3 as central protein.

### Extraction of genes associated with essential hypertension from the literature

We searched candidate genes associated with EH by PolySearch text mining system, which can produce a list of concepts relevant to the user’s query by analyzing multiple information sources including PubMed, OMIM, DrugBank and Swiss-Prot. It covers many types of biomedical concepts including diseases, genes/proteins, drugs, metabolites, SNPs, pathways and tissues [8]. We used PolySearch system to search the genes associated with EH. The query type is ‘Disease-Gene/Protein Association’ and the query keyword is ‘essential hypertension’. PolySearch system returns 1435 literatures. To check the accuracy, we manually confirmed whether these genes are associated with the essential hypertension. Finally a total of 69 candidate genes were obtained (Table 1).

**Table 1:** The list of genes extracted from literary database showing association with essential hypertension

**SN**	**Symbol**	**Description**
1	ACE2	angiotensin I converting enzyme 2
2	ADD1	adducin 1
3	ADIPOQ	adiponectin, C1Q and collagen domain containing
4	ADRA2B	adrenergic, alpha-2B-, receptor
5	ADRB1	adrenergic, beta-1-, receptor
6	ADRB2	adrenergic, beta-2-, receptor, surface
7	AGT	angiotensinogen
8	AGTR1	angiotensin II receptor, type 1
9	ALDH2	aldehyde dehydrogenase 2 family
10	ATP1A1	ATPase, Na+/K + transporting, alpha 1 polypeptide
11	ATP1B1	ATPase, Na+/K + transporting, beta 1 polypeptide
12	BDKRB2	bradykinin receptor B2
13	CACNA1C	calcium channel, voltage-dependent, L type, alpha 1C subunit
14	CACNA1D	calcium channel, voltage-dependent, L type, alpha 1D subunit
15	CALCA	calcitonin-related polypeptide alpha
16	CHGA	chromogranin A
17	CLCNKB	chloride channel Kb
18	CYBA	cytochrome b-245, alpha polypeptide
19	CYP11B2	cytochrome P450, family 11, subfamily B, polypeptide 2
20	CYP19A1	cytochrome P450, family 19, subfamily A, polypeptide 1
21	CYP2J2	cytochrome P450, family 2, subfamily J, polypeptide 2
22	CYP4A11	cytochrome P450, family 4, subfamily A, polypeptide 11
23	CYP4F2	cytochrome P450, family 4, subfamily F, polypeptide 2
24	EMILIN1	elastin microfibril interfacer 1
25	FABP3	fatty acid binding protein 3
26	FBN1	fibrillin 1
27	FURIN	furin (paired basic amino acid cleaving enzyme)
28	GDF15	growth differentiation factor 15
29	GNB3	guanine nucleotide binding protein, beta polypeptide 3
30	GRK4	G protein-coupled receptor kinase 4
31	GSTM3	glutathione S-transferase mu 3 (brain)
32	GUCA2B	guanylate cyclase activator 2B
33	HMOX1	heme oxygenase 1
34	HSD3B1	hydroxy-delta-5-steroid dehydrogenase, 3 beta- and steroid delta-isomerase 1
35	HSPA4	heat shock 70 kDa protein 4
36	IL10	interleukin 10
37	IL6	interleukin 6
38	KL	klotho
39	KLK1	kallikrein 1
40	KLKB1	kallikrein B, plasma 1
41	KNG1	kininogen 1
42	MTHFR	5,10-methylenetetrahydrofolate reductase
43	NEDD4L	neural precursor cell expressed, developmentally down-regulated 4-like
44	NOS3	nitric oxide synthase 3
45	NPPB	natriuretic peptide precursor B
46	NPR1	natriuretic peptide receptor A/guanylate cyclase A
47	NR3C2	nuclear receptor subfamily 3, group C, member 2
48	P2RY2	purinergic receptor P2Y, G-protein coupled, 2;
49	PNMT	phenylethanolamine N-methyltransferase
50	PPARG	peroxisome proliferator-activated receptor gamma
51	PSMA6	proteasome subunit, alpha type, 6
52	PTGER2	prostaglandin E receptor 2
53	PTK2B	PTK2B protein tyrosine kinase 2 beta
54	PTPN1	protein tyrosine phosphatase, non-receptor type 1
55	REN	renin
56	RNLS	renalase, FAD-dependent amine oxidase
57	SCNN1A	sodium channel, nonvoltage-gated 1 alpha
58	SCNN1B	sodium channel, nonvoltage-gated 1, beta
59	SELE	selectin E
60	SLC6A9	solute carrier family 6, member 9
61	SLC7A1	solute carrier family 7, member 1
62	SLCO1B1	solute carrier organic anion transporter family, member 1B1
63	SOD3	superoxide dismutase 3, extracellular
64	TGFB1	transforming growth factor, beta 1
65	TH	tyrosine hydroxylase
66	TNFRSF4	tumor necrosis factor receptor superfamily, member 4
67	VEGFA	vascular endothelial growth factor A
68	WNK1	WNK lysine deficient protein kinase 1
69	WNK4	WNK lysine deficient protein kinase 4

### Scanning protein-protein interactions

The candidate genes listed in Table 1 were converted to be the seed proteins. We obtained PPIs from STRING database, a precomputed database for the exploration of protein–protein interactions. The newest version of STRING, 9.0, covers approximately 2.5 million proteins from 630 different organisms [9].

### Construction of PPIs network and extracting the giant component from the extended network

We constructed an extended network that not only consists of the seed proteins but their direct PPI neighbors and the interactions between these proteins. The network was constructed using Pajek [10], a highly versatile program for the analysis, operation and visualization of large networks. In this study, the extended network includes a giant component and two small separate components derived from two seeds proteins. This study aimed to explore the mechanism of EH at the system level and the nodes with large BC value must be in the giant network obviously because both of two small separate components consist of small number of nodes, so only the giant network and its parameters related to the network theory had been analyzed or processed. In order to analyze and process the giant network conveniently, we extracted it from the extended network.

### Topological analysis of protein interaction network

Properties of nodes including connectivity degree (k), betweenness centrality (BC) and closeness centrality (CC) were adopted to evaluate the nodes in a network; especially k and BC are two fundamental parameters in the network theory [6,11]. Degree (k), the most basic characteristic of a node in a network is defined as the number of adjacent links, i.e. the number of interactions that connect one protein to its neighbors. BC is the fraction of the number of shortest paths that pass through each node, which measures how often nodes occur on the shortest paths between other nodes. The shortest path is calculated by measuring the length of all the geodesics from or to the vertices in the network. A node with high BC has great influence over what flows in the network. BC may play a major role as a global property since it is a useful indicator for detecting bottlenecks in a network. Closeness centrality (CC) is defined as the inverse of the average length of the shortest paths to/from all the other nodes in the graph, which tells us the topological center of the network. Global topological measurements of networks include average degree, mean shortest path length and diameter used to character network [6]. Average degree (<k>): it represents the mean of all degree values of nodes in a network. Mean shortest path length (mspl): is the average of the steps needed to connect every pair of nodes through their shortest path. Diameter (D): is the longest among all shortest paths. In this study, properties of nodes and measurements used to characterize network were calculated by Pajek software.

### Searching high BC nodes to create a backbone network

In this study, we viewed PPI maintaining the blood pressure homeostasis as a transportation network. Thus, the proteins with high BC should be the heavily used intersections, these proteins and the links between them make up a backbone network. The critical point of high BC was set at 5% of the total node set of the network [12,13]. These high BC nodes and the links between them were extracted from the giant network to create a backbone network. BC was originally introduced to measure the centrality of the nodes in a network. By definition, most of the shortest paths in a network go through the nodes with high BC. These nodes function as bottleneck control the communication among other nodes in the network.

### Construction a subnetwork consisting of all shortest paths between the candidate genes

Even in the giant network, there are a few pairs of candidate gene are not connected directly. In order to construct a subnetwork in which all genes associated with EH are connected directly or indirectly with minimum number of nodes, we found out all shortest paths between every pair of candidate genes. The shortest paths between the candidate genes are calculated by Pajek software. Then the subnetwork consists of nodes in these paths.

### Validation of the backbone network and the NOS3 as central protein

In order to validate the robustness of the backbone network and the NOS3 as central protein, we constructed test networks only using a part of 69 genes as initial seeds. The initial seed genes were determined by omitting from 1 to 7 (10% of 69) genes. If the number of the omitted genes is 3, there are 314364 (69 × 68 × 67) combinations. Therefore, the omitted genes were selected randomly if the number of omitted genes is more than 3. However, considering the importance of NOS3 in our conclusion, NOS3 was omitted always. Then the exact method of omitting genes is as below. If the number of omitted gene is 1, then there are 69 combinations because every gene of 69 genes was omitted once. If the number of omitted gene is 2, then there are 68 combinations (NOS3 and other 68 genes). If the number of omitted genes is more than 3, the omitted genes are NOS3 and other genes selected randomly and regardless of number of omitted genes, randomly selecting is 30 times. Finally, 287 test networks (69 + 68 + 5*30) ware constructed (Additional file 1), and the BC values of nodes in these networks ware calculated by Pajek software. Then the nodes with top 27 BC value were determined in these test networks. We tested the robustness of the backbone network and the NOS3 as central protein by calculating frequency of NOS3 as a node with the largest BC value and the accuracy of the backbone nodes in the test networks. The accuracy of backbone was estimated as the fraction of the nodes with top 27 BC in the test networks which agree with the nodes in the backbone network described in step five.

## Results

### Protein-protein interaction network

**Figure 1:**
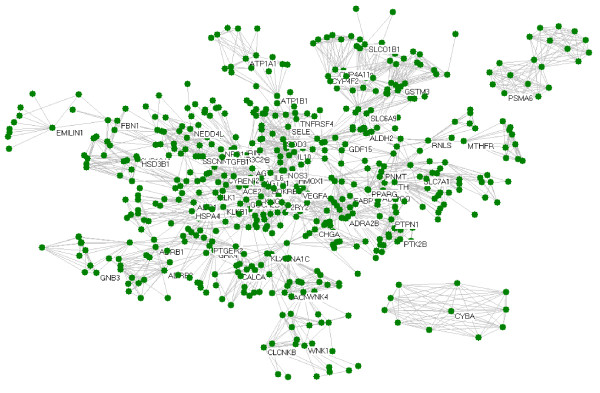
**Overview of the extended network.** The extended network includes one giant network and two separated small networks which are derived respectively from the seed protein CYBA (cytochrome b-245, alpha polypeptide) and PSMA6 (proteasome subunit, alpha type, 6). The nodes with label are seed proteins converted from the candidate genes listed in Table 1 while the nodes without name are their neighbors scanned from STRING database.

**Figure 2:**
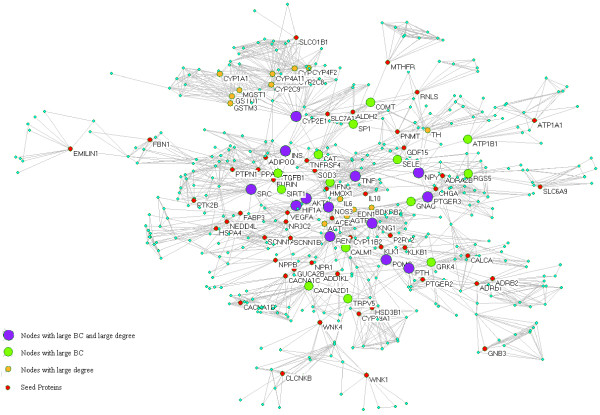
**The topology of the giant network.** The giant network extracted from the extended network is the biggest component in the extended network. The size of nodes corresponds to their BC values.

**Figure 3:**
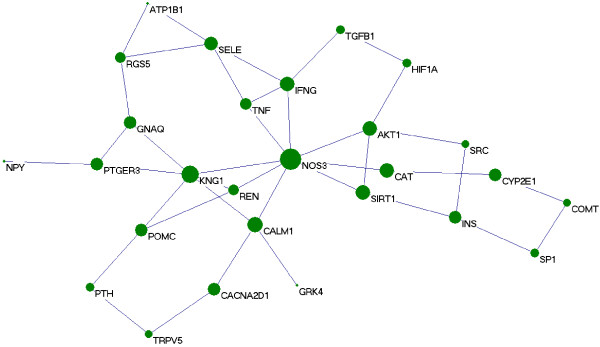
**The topology of the backbone network.** The backbone network consists from 27 nodes with high BC value. The size of nodes corresponds to their BC values.

The extended network includes one giant network and two separated small networks which are derived respectively from the seed protein CYBA (cytochrome b-245, alpha polypeptide) and PSMA6 (proteasome subunit, alpha type, 6) (Figure 1). The giant network consisted of 535 nodes connected via 2572 edges (Figure 2). The backbone network consisted of 27 nodes connected via 39 edges (Figure 3). Accordingly, we studied the measurements charactering network listed in Table 2: number of nodes (N), average degree (<k>), diameter (D) and mean shortest path length (mspl). The largest degree in the giant network is 43, while its average degree is 7.61. This network is characterized by a small number of highly connected nodes, while most of the other nodes have few connections. It indicates that the giant network is similar to other human PPIs [14].

**Table 2:** The general network measurements for networks

**Symbol**	**Description**	**Giant network**	**Backbone network**
N	number of nodes	535	27
<K>	average degree	7.61	2.89
D	diameter	12	6
mspl	mean shortest path length	5.23	3.15

### Key nodes in the PPI network

In this study, the nodes with large degree or high BC were viewed as key nodes, and 5% of the total nodes set of the network was used as the critical point of large degree and high BC nodes. Of 535 total nodes, 27 nodes have high BC (Table 3), 28 nodes have large degree (Table 4) and 13 nodes were selected with high BC and large degree (Table 5) and 14 nodes only with high BC (Table 6). In order to discern their roles in the network, these nodes were highlighted in different color and size (Figure 2). KNG1 (kininogen 1) is a hub protein with the largest degree, while NOS3 (nitric oxide synthase 3) is a bottleneck protein with the highest BC. NOS3 has highest CC value, which indicates that NOS3 locates at the centre of the network.

**Table 3:** The list of high BC nodes and their CC values

**SN**	**Symbol**	**BC**	**CC value**	**SN**	**Symbol**	**BC**	**CC value**
1	NOS3	0.27867	0.3117*	15	SIRT1	0.07233	0.2581
2	CAT	0.16556	0.2592	16	IFNG	0.07148	0.2671
3	CYP2E1	0.15796	0.2273	17	RGS5	0.06577	0.2244
4	CALM1	0.15583	0.2763	18	SRC	0.05782	0.2356
5	KNG1	0.15308	0.2985	19	GRK4	0.05735	0.2246
6	NPY	0.10990	0.2526	20	TRPV5	0.05700	0.2170
7	HIF1A	0.09982	0.2657	21	AKT1	0.05543	0.2727
8	POMC	0.09969	0.2570	22	ATP1B1	0.05506	0.2005
9	REN	0.09478	0.2809	23	SELE	0.05447	0.2313
10	TNF	0.08741	0.2645	24	GNAQ	0.05290	0.2569
11	PTH	0.08115	0.2385	25	PTGER3	0.05178	0.2542
12	COMT	0.07950	0.2158	26	SP1	0.04688	0.2198
13	TGFB1	0.07518	0.2426	27	INS	0.04607	0.2303
14	CACNA2D1	0.07511	0.2284				

*: NOS3 has the highest CC value.

**Table 4:** The list of large degree nodes and their CC values

**SN**	**Symbol**	**Degree**	**CC value**	**SN**	**Symbol**	**Degree**	**CC value**
1	KNG1	43	0.2985	15	PTH	20	0.2385
2	TNF	37	0.2645	16	AKT1	19	0.2727
3	REN	33	0.2809	17	HIF1A	19	0.2657
4	CYP2E1	28	0.2273	18	POMC	19	0.2570
5	NOS3	25	0.3117	19	CYP2J2	18	0.1875
6	AGTR1	23	0.2786	20	IL6	18	0.2671
7	CYP2C9	23	0.1886	21	CYP2C8	17	0.1880
8	EDN1	23	0.2723	22	CYP4A11	17	0.1875
9	NPY	23	0.2526	23	CYP4F2	17	0.1874
10	SRC	23	0.2356	24	GSTM3	17	0.1876
11	BDKRB2	21	0.2734	25	GSTO1	17	0.1876
12	AGT	20	0.2767	26	MGST1	17	0.1876
13	CYP1A1	20	0.1603	27	PTGER3	17	0.2542
14	INS	20	0.2303	28	TH	17	0.2208

**Table 5:** The list of proteins with both high BC and large degree and their functions

**Symbol**	**Function description**
NOS3	Produces nitric oxide which is implicated in vascular smooth muscle relaxation.
CYP2E1	An effective producer of reactive oxygen species.
KNG1	Precursor of vasoactive kinins.
NPY	A peptide with direct and potential effects on vasoconstriction.
HIF1A	Functions as a master transcriptional regulator of the adaptive response to hypoxia.
POMC	Controls energy homeostasis.
REN	Generates angiotensin I from angiotensinogen in the plasma.
TNF	A proinflammatory cytokine which induce endothelial dysfunction.
PTH	Elevates calcium level by dissolving the salts in bone and preventing their renal excretion.
SRC	Involves in cell maintenance and communication.
AKT1	Phosphorylates NOS3.
PTGER3	Receptor for prostaglandin E2.
INS	Decreases blood glucose concentration.

**Table 6:** The list of proteins only with high BC and their functions

**Symbol**	**Function description**
CAT	Protects cells from the toxic effects of hydrogen peroxide.
COMT	Inactivates catecholamine neurotransmitters and catechol hormones.
CALM1	Activates NO synthesis in NOS3.
TGFB1	Controls proliferation, differentiation and other functions in many cell types.
CACNA2D1	Calcium channel, voltage-dependent, alpha 2/delta subunit 1.
SIRT1	NAD-dependent protein deacetylase.
IFNG	Has an important immunoregulatory function.
RGS5	A potent GTPase-activating protein for Giα and Gqα.
GRK4	Specifically phosphorylates the activated forms of G protein-coupled receptors.
TRPV5	Actives calcium selective cation channel involved in Ca (2+) reabsorption.
ATP1B1	Maintains the normal gradients of Na (+) and K (+) across plasma membrane.
SELE	Cell-surface glycoprotein having a role in immunoadhesion.
GNAQ	Acts as an activator of phospholipase C.
SP1	Activates or represses transcription in response to stimuli.

### The signaling pathsway in the high BC network and cross-talk between them derived from backbone network

The backbone network consists from 27 high BC nodes, the size of which corresponds to their BC value and the 39 links between them (Figure 3). Without calculating the values of BC and CC, we can find out that NOS3 locates at the centre of the backbone network with the highest BC value and the largest degree. NOS3 has 8 neighbors: SIRT1, CAT, AKT1, IFNG, TNF, KNG1, REN and CALM1. These proteins also represented SIRT1 pathway, antioxidant system, AKT pathway, inflammatory system, kallikrein-kinin system, rennin-angiotensin system and Calcium signaling pathway. The details of other proteins in the backbone network were not presented here.

### Subnetwork consisting of all shortest paths between the candidate genes

**Figure 4:**
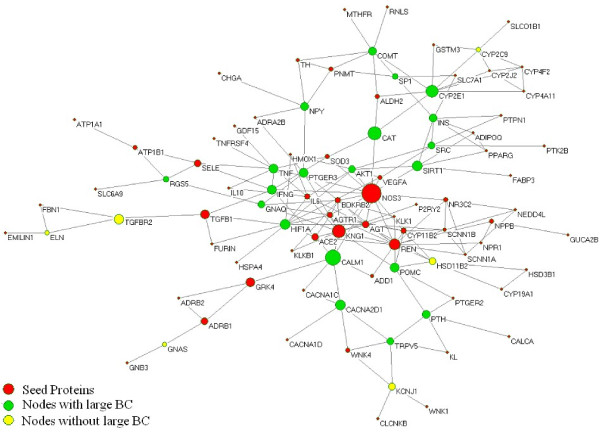
**The subnetwork consisting of all shortest paths between the genes associated with essential hypertension.** The candidate genes are connected by all shortest paths in the giant network. The size of nodes corresponds to their BC values and there are 6 yellow nodes without large BC (6 outside 27).

This subnetwork consists of 93 nodes including 6 proteins which are not large BC nodes nor seed proteins, 60 seed proteins, 20 large BC nodes and 7 nodes which are both seed protein and large BC node (Figure 4). We can find out that NOS3 has the highest BC value and the top 27 BC nodes in this subnetwork coincide well with 27 nodes in the backbone network. There are only 6 proteins is not in the list of 27 nodes with large BC value in the giant network. They are TGFBR2, AGT, ACE2 GNAQ, HSD11B2 and KCNJ1 (Table 7).

**Table 7:** The list of top 27 BC nodes in the subnetwork consisting of candidate genes mainly

**SN**	**Symbol**	**BC**	**SN**	**Symbol**	**BC**
1	NOS3	0.407569711	15	TNF	0.075270436
2	CALM1	0.235872425	16	COMT	0.069978479
3	KNG1	0.177579326	17	TGFBR2	0.063784042
4	CAT	0.176160022	18	NPY	0.062641811
5	CYP2E1	0.146210287	19	PTH	0.059707133
6	REN	0.142239722	20	INS	0.058584741
7	CACNA2D1	0.105749149	21	AGT	0.052421668
8	SIRT1	0.100505648	22	TRPV5	0.050090109
9	HIF1A	0.09411956	23	ACE2	0.049687235
10	POMC	0.093076135	24	AKT1	0.048366969
11	TGFB1	0.087628641	25	GNAQ	0.047654533
12	GRK4	0.084089823	26	HSD11B2	0.04414159
13	PTGER3	0.082678839	27	KCNJ1	0.043239369
14	IFNG	0.082446095			

### The robustness of the backbone network and the NOS3 as central protein

**Figure 5:**
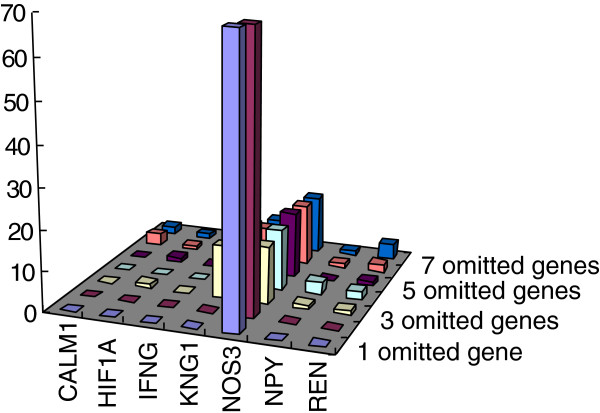
The frequency of the nodes with the largest BC value in the test networks grouped by the number of omitted genes.

**Figure 6:**
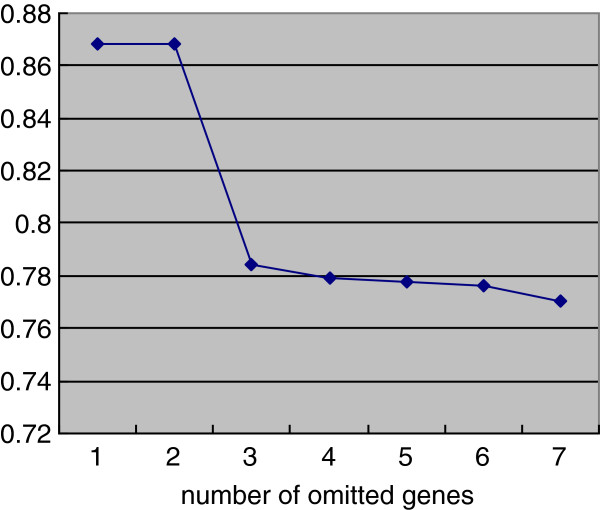
The accuracy of the backbone grouped by the number of the omitted genes.

There are 7 genes with the largest BC value in the test networks. They are CALM1, HIF1A, IFNG, KNG1, NOS3, NPY and REN (Additional file 1). Though NOS3 is not as the initial seed gene, its frequency as the node with the largest BC value is 211 in 287 test networks (Table 8 and Figure 5). The accuracy of the backbone is 0.80344 (Table 8). Both the accuracy of the backbone and the frequency of NOS3 as the node with the largest BC value decrease rapidly when the number of omitted genes is 3 (Table 8 and Figure 6).

**Table 8:** Frequency of nodes with the largest BC value and accuracy of backbone in the 287 test networks

**Number of omitted genes**	**Frequency of nodes with the largest BC value in the test networks**							**Accuracy of the backbone**	**Number of the test networks**
	**CALM1**	**HIF1A**	**IFNG**	**KNG1**	**NOS3**	**NPY**	**REN**		
1	0	0	0	0	69	0	0	0.86795	69
2	0	0	0	0	68	0	0	0.86849	68
3	0	1	0	13	14	1	1	0.78395	30
4	0	0	0	10	15	3	2	0.77901	30
5	0	1	0	12	16	0	1	0.77778	30
6	3	1	0	8	15	1	2	0.77654	30
7	2	1	1	7	14	1	4	0.77037	30
Summary	5	4	1	50	211	6	10	0.80344	287

## Discussion

Though larger number of study had been finished on EH and many casual or susceptible genes related to EH had been reported, its pathogenesis remains elusive. We proposal that the proteins encode by these genes can determine BP level by the interactions between them. The purpose of this study is to analysis the contribution of these proteins to the pathogenesis of EH and discovers other key proteins cooperating with them by topological analyses. As two fundamental measures in the network theory, degree and betweenness had been widely used to evaluate the proteins in the different PPIs associated with diseases, though there are some new parameters derived from them [12,28-30]. We also utilized degree and betweenness as main parameters to evaluate the nodes in the PPIs.

In this study, 69 genes have been searched as causative or susceptible genes involved in EH. The network derived from seed proteins converted from these genes, consists a giant network and two separated small network (Figure 1). Only two seed proteins (CYBA and PSMA6) separate from the giant network, it suggests that the PPIs between these proteins orchestrate the BP variation. There must be some missed genes from literature searching and new causative or susceptible genes remained to be discovered for EH, even false nodes result from false interactions in the network. However, as reviewed by Gipsi Lima-Mendez and Jacques van Helden [14], biological networks are tolerant to nodes deletion, and new nodes prefer to link to nodes with large degree. In another word, biological networks are robust to random alteration of nodes but sensitive to hub removal.

In the giant network, there are 28 proteins with large degree and 27 proteins with high BC, 13 proteins with both large degree and high BC among them (Tables 3, 4, 5 and Figure 2). In order to disentangle the effects of betweenness and degree, Yu and co-workers divided all proteins in a certain network into four categories [7]: nonhub–nonbottlenecks (small degree and low BC); hub–nonbottlenecks (large degree but low BC); nonhub–bottlenecks (small degree but high BC); and hub–bottlenecks (large degree and high BC). Han et al. distinguish two subtypes among the highly connected proteins: hub–bottlenecks tend to be date-hubs, whereas hub–nonbottlenecks tend to be party-hubs. Party hubs interact with most of their partners simultaneously, whereas date hubs bind different partners at different times or locations [15]. We believe that further verify the space-time effect of these proteins, which will help us to identify drug targets and biomarkers for EH. KNG1 with the largest degree ranks 5 in the high BC proteins list while NOS3 with the highest BC ranks 5 in the large degree proteins list. KNG1 representing kallikrein-kinin system and NOS3 representing Endothelial NO system both mainly function as vasodilatation in the regulation of BP. In certain degree, we can cautiously speculate that EH originates from the failure of systemic or local vasodilatation in the right time and right place.

NOS3 with the largest CC value locates at the centre of the giant network and the backbone network derived from high BC proteins, which highlight the significant role of NO system in maintaining BP homeostasis. In the study, the backbone network centering on NOS3 is a signaling high pathway to regulate the BP variation (Figure 3). The proteins within it are key intersections. The intersections direct linking to NOS3 include SIRT1, CAT, AKT1, IFNG, TNF, KNG1, REN and CALM1. It has been reported that SIRT1 promotes endothelial-dependent vasodilatation by targeting NOS3 for deacetylation, leading to enhance nitric oxide (NO) production [16]. A recent study has shown that production of NO, stimulated by caloric restriction, increases SIRT1 expression; this study suggests that eNOS may be involved in regulation of the expression of SIRT1 in murine white adipocytes [17]. Although H_2_O_2_ is not directly involved in NO synthesis, the H_2_O_2_/ CAT stimulate NO synthase activity [18]. As the major cardiovascular enzymatic antioxidants, CAT indicates the role of oxidative stress in the hypertension [19]. Akt regulates the activity of NOS3 via phosphorylation at Ser1177, regulating NO production and vasodilation [20]. It has been estimated that Akt kinase has over 9000 possible substrates [21]. The evidence regarding the role of inflammatory system (TNF, IFNG) and renin-angiotensin system (REN) in BP regulation and their interactivity with NOS3 is available anywhere. After release from its precursor KNG1, kinin regulates NOS3 by activating two distinct G protein-coupled receptors called B2R and B1R [22]. CALM1 activates NO synthesis in NOS3 through a conformation change of the flavin mononucleotide domain from its shielded electron-accepting state to a new electron-donating state [23]. Theses proteins also represented SIRT1 pathway, antioxidant system, AKT pathway, inflammatory system, kallikrein-kinin system, rennin-angiotensin system and Calcium signaling pathway. Their role in BP regulation and their interactions with NO system are reported by many researches [23-27].

The backbone network presents a clear and visual overview which shows all important genes and related regulatory pathways for BP and the crosstalk between them. In order to further confirm the role of NOS3 and other proteins in the backbone network, we construct a subnetwork consisting of all shortest paths between the candidate genes (Figure 4). In this subnetwork there are only 6 proteins neither seed proteins converted from candidate genes or nodes with large BC value in the giant network. In another word, the large BC nodes can connect and integrate these seed proteins well. We can also find out that NOS3 has the highest BC value and the top 27 BC nodes in this subnetwork coincide well with 27 nodes with large BC value in the giant network.

To test how robust the conclusions obtained in this work against the change of initial seed genes, 287 test networks had been constructed by omitting several initial seed genes. Despite that NOS3 was not as initial seed genes always, its frequency as a node with the largest BC value is 211 in 287 test networks. KNG1, REN, NPY, CALM1, HIF1A and IFNG Flowing NOS3, their frequency is 50, 10, 6, 5, 4 and 1 respectively (Table 8, Figure 5). All of these 7 proteins are the nodes with high BC and degree in the original network (Table 5). The accuracy of backbone is 0.80344 (Table 8). Both the accuracy of the backbone and the frequency of NOS3 as the node with the largest BC value decrease rapidly when the number of omitted genes is 3 (Table 8 and Figure 6). It may suggest that the NOS3 as central protein and the component of backbone network dependent each other.

## Conclusion

Most of seed proteins (67 of 69) associated with EH and their PPI neighbours connected to a giant network. The backbone network presented a clear overview, which shown all important genes, their related regulatory pathways for BP and the crosstalk between them. The backbone network is robust against the changes of initial seed genes. Our finding suggested that blood pressure variation was orchestrated by an integrated PPI network centered on NOS3.

## Competing interests

The author(s) declare that they have no competing interests.

## Authors’ contributions

RJ made substantial contributions to conception and design, construction of PPIs, analysis of PPIs, was involved in drafting the manuscript or revising it critically for important intellectual content, and gave final approval of the version to be published. LH made substantial contributions to construction of PPIs, analysis of PPIs, was involved in drafting the manuscript or revising it critically for important intellectual content, and gave final approval of the version to be published. FJ made substantial contributions to searching and validation of genes related to essential hypertension, and gave final approval of the version to be published. LL made substantial contributions to searching and validation of genes related to essential hypertension, and gave final approval of the version to be published. XY made substantial contributions to searching and validation of genes related to essential hypertension, and gave final approval of the version to be published. LX made substantial contributions to Scanning protein interactions from the database STRING and gave final approval of the version to be published. SH made substantial contributions to Scanning protein interactions from the database STRING. CY made substantial contributions to Scanning protein interactions from the database STRING. JX made substantial contributions to Scanning protein interactions from the database STRING. LY made substantial contributions to searching and validation of genes related to essential hypertension, and gave final approval of the version to be published. LH made substantial contributions to conception and design, construction of PPIs, analysis of PPIs, was involved in drafting the manuscript or revising it critically for important intellectual content, and gave final approval of the version to be published. All authors read and approved the final manuscript.

## Supplementary Material

Additional file 1 Detail information for frequency of nodes with the largest BC value and accuracy of backbone in the 287 test networks. Click here for file
